# Comprehensive Evaluation of Physicochemical Parameters in Retail Chicken Meat

**DOI:** 10.3390/foods14244276

**Published:** 2025-12-12

**Authors:** Ángela Serrano Ayora, Carmen Avilés-Ramírez, Rosa M. García-Valverde, Andrés L. Martínez Marín

**Affiliations:** 1 Departamento de Producción Animal, Universidad de Córdoba, Ctra. Madrid-Cádiz km 396, 14071 Córdoba, Spain; v72seaya@uco.es (Á.S.A.); pa1martm@uco.es (A.L.M.M.); 2 Departamento de Bromatología y Tecnología de los Alimentos, Universidad de Córdoba, Ctra. Madrid-Cádiz km 396, 14071 Córdoba, Spain; v62gavar@uco.es

**Keywords:** poultry, breast, quality, lipids, volatile compounds

## Abstract

The aim of the present study was to characterize the chemical and quality traits of retail chicken meat in Spain. A total of 39 breast (*Pectoralis major*) samples were collected from large stores across three seasons in 2024 (13 samples per season). All samples were consistently sourced from the same 13 suppliers, that collectively account for more than 70% of Spain’s broiler production. Based on retail label claims, samples were grouped as either ‘non-certified’ (no claims; 7 samples per season) or ‘certified’ (certified claims regarding distinctive dietary and slaughter age practices; 6 samples per season). Proximate composition, quality traits (pH, color, water-holding capacity, texture, oxidative stability), and the profiles of fatty acids (FAs) and volatile organic compounds (VOCs) were analyzed. Meat from the certified group had a higher protein content (22.37% vs. 20.62%; *p* < 0.01) and lower thawing (3.22% vs. 6.59%; *p* < 0.001) and cooking losses (14.09% vs. 24.64%; *p* < 0.01). Certified meat was also darker (lower L*: 48.48 vs. 52.59; *p* < 0.05) and exhibited a more intense yellow color (higher b*: 18.66 vs. 4.22, hue angle: 87.63 vs. 66.59, and chroma: 18.71 vs. 4.62; all *p* < 0.001). The intramuscular fat of certified meat contained less monounsaturated FAs (34.72% vs. 40.32%; *p* < 0.001) and more polyunsaturated FAs (28.82% vs. 23.55%; *p* < 0.001). Eight of the thirteen nutritional indices derived from the FAs profile were more favorable in the certified group. A total of 171 VOCs were identified, with sulfur compounds being more abundant in certified meat (0.94% vs. 0.67%; *p* < 0.05). In conclusion, retail chicken meat grouped according to commercial labeling possesses a distinct chemical and quality profile.

## 1. Introduction

Global consumption of poultry meat has increased steadily in recent decades (from 7.65 kg per capita in 1990 to 17.38 kg in 2023), largely due to its affordability, nutritional profile, and broad cultural acceptance [1,2]. Poultry now accounts for nearly 40% of total meat production worldwide (144 out of 370 million tonnes), with broiler chickens (*Gallus gallus domesticus*) dominating the sector (87.5% of total poultry meat) [1]. This expansion is driven by low production cost, high biological efficiency, and the absence of significant cultural or religious barriers compared with other meats [3,4].

In Spain, poultry meat is the most consumed fresh meat (11.97 out of 29.64 kg per capita) [5], and the value of poultry meat production (84% chicken meat) accounts for the third largest livestock sector after beef and pork (11.7% vs. 13.9% and 41.3% of gross agricultural output, respectively) [6]. The sector is primarily based on an intensive conventional system, characterized by high stocking densities, fast-growing genetic lines and advanced technological inputs [7,8]. In contrast, alternative production systems (e.g., certified, organic, free-range) are typically defined by lower stocking densities, the use of slow-growing genotypes slaughtered at an older age, and specific dietary regimens [8]. Within the European Union, labeling claims regarding these distinctive production practices are permitted for poultry suppliers, provided they comply with the criteria established under Regulation (EC) No 543/2008 [9]. Although still limited in scale, alternative production systems address consumer concerns regarding sustainability, animal welfare, and product differentiation [10,11,12].

Meat quality is multi-faceted, determined by intrinsic attributes such as chemical composition, lipid profile, and quality traits, as well as by extrinsic factors including production system and environmental footprint [13,14]. These factors directly influence consumer perception at purchase and consumption. For instance, color is a primary indicator of freshness [15], water-holding capacity (WHC) affects juiciness and tenderness [16], and the profiles of fatty acids (FAs) and volatile organic compounds (VOCs) determine both perceived healthiness and sensory properties [17,18]. While several studies have examined the impact of alternative production systems on those parameters [19,20], few have focused on chicken meat marketed at the retail level [21,22,23,24]. Those studies involved a limited number of suppliers, either lacked seasonal sampling or did not specify it, and did not carry out a comprehensive laboratory characterization of the meat assayed. Furthermore, the scarcity of comparative studies may hinder a robust understanding of how the attributes referenced by certified label claims influence the final product available to consumers. However, it is important to acknowledge that retail label claims do not equate to an independent verification of actual production practices nor represent homogeneous production systems, but rather commercial categories. Therefore, comparisons based on such labels reflect commercial categories rather than verified or uniform production systems.

The objective of the present study was to compare the chemical composition and quality traits of retail chicken meat based solely on label-defined groups (‘non-certified’ vs. ‘certified’ claims) in the Spanish market.

## 2. Materials and Methods

### 2.1. Experimental Design

A total of 39 breast (*Pectoralis major*) samples were collected from large stores across three seasons in 2024 (13 samples per season). Seasonal sampling was only intended to increase the variability within suppliers. No samples were collected during summer due to extreme seasonal temperatures and institutional closure. We confirmed that all samples originated always from the same 13 suppliers based on the information provided on the labels. Those 13 suppliers collectively account for more than 70% of Spain’s broiler production [25]. Based on label claims, samples were grouped as either ‘non-certified’ (no claims; 7 samples per season) or ‘certified’ (certified claims regarding distinctive dietary and slaughter age practices; 6 samples per season). Label claims were certified by a certification body (SGS or Certicar companies) in accordance with Commission Regulation (EC) No 543/2008 [9]. All the six suppliers in the certified group declared that cereals accounted for at least 65% by weight of the feed formula. Additionally, five of them also specified that maize accounted for at least 50% of the feed, three stated a minimum slaughter age of 56 days, and one additionally indicated ‘free range’ raising conditions. It is essential to clarify that all sample comparisons in this study were based strictly on the retail label claims and not on independent verification of the actual production systems. Moreover, lack of homogeneity in production practices between suppliers within both groups was expected. All products were purchased within standard expiration ranges (mean 2.8 ± 1.5 days post-packaging) to ensure comparable storage conditions. Samples were transported refrigerated (2–4 °C) to the Food Technology Laboratory, University of Córdoba, and processed within 24 h.

### 2.2. Laboratory Analyses

Moisture, protein, fat, and ash were determined following AOAC methods [26]: oven-drying at 100–102 °C for at least 16 h (AOAC 950.46), Kjeldahl (N × 6.25; AOAC 981.10), Soxhlet extraction (AOAC 960.39), and muffle incineration at 550 °C ± 25 °C for 4–6 h (AOAC 920.153), respectively.

The pH value, color, WHC, texture, and lipid oxidation were measured. pH was determined in triplicate with a Hanna Edge HI2020-48 pH meter (Hanna Instruments, Eibar, Spain) equipped with a penetration electrode. Color parameters (L*, a*, b*) were recorded after 30 min of blooming using a Minolta CR-400 colorimeter (Minolta Co., Osaka, Japan). Illuminant D65, an 8 mm measurement aperture, and a 2° standard observer were selected as measurement conditions. The hue angle and chroma values were determined using the following formulae:

(1) $$Hue\;angle={tan}^{-1}[b^{*}/a^{*}]$$

(2) Chroma=a*2+b*212

WHC was analyzed by measuring expressible moisture as well as thawing, cooking, and drip losses [27,28,29,30,31]. In brief, for expressible moisture determination, 5 g of sample were excised, weighed, wrapped in pre-weighed Whatman No. 1 filter paper, and placed between two plates. A constant load of 2500 g was applied for 5 min. The moisture absorbed by the filter paper after compression was used to calculate the percentage of expressible moisture. Thawing loss determination, breast meat samples were weighed before thawing and again after gently blotting surface moisture post-thaw. Thaw loss was calculated as the percentage difference between the weights before and after thawing. For cooking loss, weighed breast meat samples were placed in a polyethylene bag and immersed in a water bath at 75 °C until reaching 70 °C internally, measured by a thermocouple placed in the geometric center. Following 30 min cooling at room temperature, samples were re-weighted and cooking loss was expressed as the percentage reduction in weight between raw and cooked samples after blotting them gently with filter paper. Finally, approximately 20 g of meat sample was suspended on a hook inside a sealed container, ensuring no contact with the walls. After 24 h of storage at 4 °C, the sample was gently blotted and weighed to determine drip loss, expressed as the percentage reduction in weight before and after storage.

Texture (shear properties) was measured by Meullenet–Owens Razor Shear (MORS) [32,33]. A TA-XT Texture Analyzer (5 kg load cell, 10 mm/s crosshead speed, 20 mm penetration; Texture Analyser, Stable Micro Systems, Surrey, UK) was used equipped with a 24 × 8.9 mm razor blade replaced every 50 tests to maintain sharpness. Shear force (RBF) was recorded as the peak force, and shear energy (RBE) as the area under the force–deformation curve.

Oxidative stability was evaluated by thiobarbituric acid-reactive substances (TBARS) by homogenizing approximately 2 g of partially chopped breast meat with 10 mL cold 17.5% trichloroacetic acid, followed by centrifugation (4500 rpm, 4 °C, 5 min) and filtration into 15 mL tubes. Samples were then mixed with 1 mL of 0.02 M TBA and incubated at 20 °C for 20 h alongside a reagent blank. Absorbance was measured at 532 nm (Helios spectrophotometer, Thermo Scientific, Bremen, Germany), and a calibration curve was constructed using 1,1,3,3-tetra-ethoxypropane (MDA) standards processed under identical conditions. MDA content was calculated as mg/kg of meat using the standard curve and standard conversion equations [34,35,36].

Intramuscular fat was extracted from 1 g of breast muscle, methylated to FAs methyl esters (FAME) [37], and analyzed by GC-FID using an Agilent 6890N Network GC System (Agilent, Inc., Santa Clara, CA, USA) equipped with a flame ionization detector (FID) and an HP-88 capillary column (100 m × 0.25 mm i.d., 0.2 μm film thickness; Agilent Technologies Spain, S.L., Madrid, Spain). The chromatographic conditions were the same as those reported by Gutiérrez-Peña et al. [38]. FAs peaks were identified against Supelco 37 Component FAME Mix (CRM47885; Supelco, Bellefonte, PA, USA), and appropriate FAs indexes were then calculated [39,40].

VOCs were analyzed after standardized cooking (170 °C to 70 °C internal temperature). After grilling, 10 g of the cooked sample were placed in a 100 mL headspace vial (Teledyne Tekmar, Mason, Ohio, USA). Volatile compounds were extracted using headspace solid-phase microextraction HS-SPME (DVB/CAR/PDMS fiber), separated by gas chromatography, and their mass spectra were obtained under the same equipment and conditions described by Gutiérrez-Peña et al. [38] with a VF-WAXms column (Agilent Technologies Spain, S.L., Madrid, Spain). The peak areas of the volatile compounds were integrated to determine their relative abundance. A series of n-alkanes (C5–C18, HP5080-8768, Agilent, Inc., Santa Clara, CA, USA) was analyzed under identical conditions to calculate the linear retention index (LRI) for each volatile compound. The tentative identification of each compound was based on comparison of their mass spectra with those in the National Institute of Standards and Technology (NIST, version 2.0, Gaithersburg, MD, USA) library or with previously published data [38,41]. A compound was considered properly identified if it achieved a library similarity of 70% or higher.

### 2.3. Statistical Analysis

Analyses were performed with SAS OnDemand for Academics 3.82 (SAS Institute Inc., Cary, NC, USA). Data were analyzed using the MIXED procedure with label-defined group, non-certified or certified, as a fixed effect and supplier nested within group as a random effect. Pearson correlations were also calculated, when appropriate. Significance was set at *p* < 0.05, with 0.05 ≤ *p* ≤ 0.10 considered trends.

## 3. Results and Discussion

### 3.1. Chemical Composition

The chemical composition of meat from both label-defined groups fell within the ranges previously reported for chicken breast [42,43]. Fat content was the most variable component, ranging from approximately 1% to 4%, consistent with earlier reports [44,45]. Breast meat composition differed between groups (Table 1). Moisture content was 1.8% higher in non-certified meat, whereas certified meat contained 8.5% more protein and tended to have lower fat content. Such results parallel the compositional profiles typically reported for slow-growing genotypes in controlled experimental studies [44,45,46,47].

### 3.2. Meat Quality Traits

Meat quality traits of the label-defined groups are summarized in Table 2. The pH values were within the normal range for fresh chicken meat [48,49], with a tendency toward higher values in certified meat. Controlled comparative studies investigating the effects of genotype, housing, and feeding have reported variable results in chicken meat pH from alternative systems, including no differences, lower, or higher values [50,51,52,53]. Such discrepancies may reflect differences in muscle glycogen reserves at slaughter [20,54,55,56]. Although storage conditions are known to affect pH [57,58], this variable should not have influenced our results, since samples from both groups were obtained under identical handling and storage conditions.

Meat WHC is relevant as it is positively associated with juiciness and consumer acceptance [59]. Our values (Table 2) fell within the ranges previously reported for chicken breast meat [52,60,61]. Certified meat exhibited markedly lower thawing and cooking losses and tended to show lower drip losses (49%, 57%, and 77% of non-certified meat values, respectively), reflecting superior WHC. This finding corresponds to outcomes noted in several controlled trials [52,62], though other studies under similar conditions reported no WHC differences [50,63,64]. WHC is known to relate inversely to pH due to effects on protein denaturation and net electrical charge [65]. However, in the present study no significant correlations were found between pH and any of the WHC measurements.

Color is a critical quality attribute for Spanish consumers [15,66]. Color was strongly different between label-defined groups (Table 2). Certified meat was darker and more yellow (lower L*, and higher b*, hue angle and chroma values), consistent with prior studies carried out under controlled conditions [23,50]. Given that xanthophylls are the primary dietary component affecting meat color [67,68], the observed differences are consistent with variations in feed composition between the commercial categories. In the present study, five of the six certified suppliers explicitly reported at least 50% maize in the diet on the label; the results suggest that the remaining supplier may have also fed a xanthophyll-enriched diet. A strong negative correlation was found between pH and L* (r = –0.562, *p* < 0.001), reflecting how post-mortem metabolism lowers pH, increases protein denaturation and light scattering, and ultimately makes the meat appear paler [58,69].

TBARS represent secondary lipid-oxidation products—primarily MDA—that form a chromogenic complex with thiobarbituric acid and are widely used as an index of oxidative deterioration in muscle foods [70]. No differences were observed for oxidative stability (TBARS) between label-defined groups (Table 2), in concordance with previous results from comparative production studies [50,71]. Despite the analytical methods could slightly affect MDA quantification, the TBARS values in our study were well below 0.8–1, which can be considered the limiting threshold for the acceptability of chicken meat [72].

The lack of differences in tenderness found in the present work (Table 2) suggests that the farming practices underlying the label claims have little impact on this trait, which generally is of limited relevance for consumers [15].

### 3.3. Intramuscular Fatty Acid Profile

The number of FAs identified and quantified in intramuscular fat (Table 3) was higher than that reported in other studies [22,62,73]. Unsaturated FAs dominated intramuscular fat in both label-defined groups, with oleic (cis-9 C18:1) and linoleic (cis-9, cis-12 C18:2, LA) acids as the major contributors, and palmitic acid (C16:0) as the main saturated fatty acid, consistent with previous reports [74].

Certified meat contained 14% less monounsaturated FAs (MUFAs), largely due to a 13.5% reduction in oleic acid (Table 3). Several studies that compared different production systems (e.g., organic, free-range and conventional) reported higher levels of MUFAs in meat samples from the conventional system [22,62,75,76], although those authors did not provide any explanation for the observed differences. In the present study, the reduction in MUFA in the certified group was accompanied by a lower C18:0 Δ9-desaturase activity index (Table 3). While direct genetic verification is beyond the scope of this retail-based study, the difference found in such index resembles metabolic variations documented between different genetic strains [77].

Total polyunsaturated FAs (PUFAs) were 22% higher in certified meat, with a 23% increase in n-6 FAs (Table 3). Particularly, LA and arachidonic acid (C20:4 n-6, ARA) increased by 13% and 93%, respectively. Moreover, docosahexaenoic acid (C22:6 n-3, DHA) was 73% higher, while α-linolenic acid (18:3 n-3, ALA) was lower in that meat. The results from previous research do not show a clear pattern in intramuscular FAs profile due to production system [73,75,76]. It is well established that the FAs composition of the diet determines the availability of preformed FAs for metabolism and, consequently, the FAs profile of chicken body fat [78,79,80]. This is particularly true for LA and ALA, which can only be obtained through dietary intake. Moreover, the deposition of long-chain PUFAs (≥ C20) in body fat depends almost entirely on the availability of LA and ALA as substrates for elongation and desaturation, as their direct supply in conventional poultry diets is minimal [81,82]. The higher proportion of LA in the intramuscular fat of certified meat aligns with the label declarations regarding feed ingredients and corresponds to findings from previous research [73]. In turn, the elongation and Δ5 + Δ6 desaturation of LA would account for the higher ARA levels observed in certified meat, supported by the higher ARA/(LA + ARA) ratio (Table 3). Similarly, the increase in DHA in the intramuscular fat of the certified group was accompanied by a higher DHA/(ALA + DHA) ratio (Table 3), which is typically indicative of enhanced elongase and desaturase activity. Boschetti et al. [77] reported higher ARA/LA and DHA/ALA ratios in slow-growing compared with fast-growing genotypes, regardless of diet.

Overall, FAs composition of certified meat resulted in more favorable nutritional indexes (Table 4), including a higher PUFAs/SFAs ratio and a 60% increase in EPA+DHA. To the best of our knowledge, direct comparisons between verified production practices regarding FAs-based nutritional indexes remain scarce. Dal Bosco et al. [39] compared slow- and fast-growing genotypes, fed the same diet and raised under the same conditions, and found more favorable values in the former.

### 3.4. Volatile Organic Compounds

**Figure 1:**
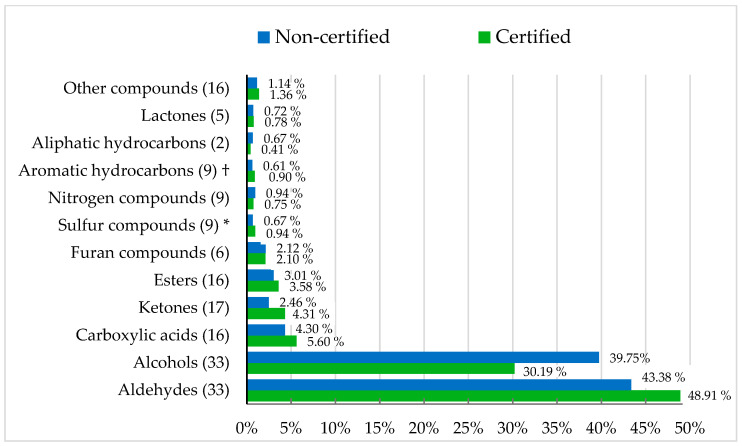
Volatile organic compound families (%) of retail chicken breast meat by label-defined group. † *p* = 0.089; * *p* < 0.05.

A total of 171 VOCs from 12 families were identified (Figure 1), exceeding the numbers previously reported in chicken meat [83,84,85]. It is well established that two distinguishing factors—genotype and slaughter age—can influence VOCs concentrations in meat [86,87,88]. However, to our knowledge, no previous studies have directly compared the VOCs profiles of meat samples obtained from controlled production system comparisons.

Label-defined group effects on VOCs profiles were subtle. Certified meat showed a greater abundance of sulfur compounds, while aromatic hydrocarbons tended to be lower than in non-certified meat. Although sulfur compounds were present at low concentrations, these molecules have very low odor thresholds and can strongly influence aroma perception [89], contributing to the characteristic ‘chicken-like’ flavor [18,90]. Only eleven individual compounds differed significantly (Table 5). Heptylbenzene and trimenal were more abundant in certified meat, whereas the other nine were more abundant in non-certified meat. Aldehydes, such as heptanal and 2,4-heptadienal, originate directly from lipid oxidation and their differences may be associated with small variations in the oxidative stability between both groups. Lipid-derived volatiles are recognized as major contributors to poultry aroma and are closely linked to fatty-acid degradation pathways [91].

Although subtle, the VOCs differences observed are important since aroma is recognized as a key attribute shaping consumer acceptance of meat [88,92]. Previous sensory studies have reported flavor differences linked to production systems [51,53,60]. The distinct VOCs patterns observed here between label-defined groups echo such reported sensory variation.

## 4. Conclusions

This study compared retail chicken breast based solely on label-defined groups, without independent verification of production practices. Despite this limitation, meat labeled as certified was associated with higher protein content, superior WHC, distinct color traits, more favorable FAs indices, and different VOCs profiles compared to non-certified meat. These differences should be interpreted as associations with labeling categories, not as direct effects of specific, verified production factors. Nevertheless, for consumers, the results obtained provide a starting point for understanding how label claims relate to meat quality. Our results also open avenues for future research under controlled experimental conditions to develop robust tools for authenticating production methods and to explore the underlying physiological mechanisms associated with these quality differences.

## Figures and Tables

**Table 1:** Chemical composition of retail chicken breast meat by label-defined group (NC: non-certified; C: certified).

Parameter	NC	C	SEM	*p*
Moisture (%)	75.99	74.65	0.178	<0.001
Ash (%)	1.41	1.46	0.042	0.506
Crude fat (%)	1.67	1.06	0.134	0.083
Crude protein (%)	20.62	22.37	0.259	<0.01
Energy value (kcal/100 g)	97.54	99.00	1.364	0.642

SEM: standard error of the mean.

**Table 2:** Meat quality traits of retail chicken breast meat by label-defined group (NC: non-certified; C: certified).

Parameters	NC	C	SEM	*p*
pH	5.76	5.89	0.035	0.052
Water holding capacity				
Thawing loss (%)	6.59	3.22	0.379	<0.001
Cooking loss (%)	24.64	14.09	1.751	<0.01
Drip loss (%)	2.22	1.72	0.150	0.095
Expressible moisture (%)	21.96	20.23	1.026	0.409
Oxidative stability				
TBARS (mg MDA/kg)	0.100	0.122	0.008	0.187
Shear measurements				
RBF (N)	10.49	10.36	0.253	0.821
RBE (N·mm)	134.35	133.29	3.151	0.888
CIELab color space indexes				
L*	52.59	48.48	0.689	<0.05
a*	1.64	0.84	0.164	0.058
b*	4.22	18.66	1.241	<0.001
Hue angle	66.59	87.63	2.238	<0.001
Chroma	4.62	18.71	1.213	<0.001

TBARS: Thiobarbituric acid reactive substances; RBF: Meullenet–Owens razor shear force; RBE: Meullenet–Owens razor shear energy; L*: lightness; a*: redness; b*: yellowness; SEM: standard error of the mean.

**Table 3:** Fatty acid profile (%) and fatty acid indexes of retail chicken breast meat by label-defined group (NC: non-certified; C: certified).

Parameters	NC	C	SEM	*p*
C8:0	0.024	0.031	0.002	0.208
C10:0	0.030	0.036	0.002	0.465
C11:0	0.024	0.028	0.002	0.501
C12:0	0.127	0.151	0.007	0.085
C13:0	0.040	0.040	0.002	0.920
C14:0	0.767	0.582	0.047	0.050
cis-9 C14:1	0.166	0.128	0.007	<0.01
C15:0	0.160	0.131	0.006	<0.05
cis-9 C15:1	0.040	0.051	0.003	0.161
C16:0	24.198	23.921	0.221	0.608
cis-9 C16:1	4.033	3.431	0.126	<0.05
C17:0	0.295	0.235	0.009	<0.01
cis-9 C17:1	0.112	0.083	0.005	<0.05
C18:0	9.918	10.684	0.180	0.069
cis-9 C18:1	34.889	30.175	0.513	<0.001
trans-9 C18:1	0.156	0.114	0.008	0.058
trans-11 C18:1 ^1^	0.213	0.158	0.008	<0.001
cis-9, cis-12 C18:2	16.702	19.462	0.356	<0.01
trans-9, trans-12 C18:2	0.070	0.067	0.005	0.766
cis-9, trans-11 C18:2	0.014	0.014	0.001	0.751
cis-10, trans-12 C18:2	0.010	0.012	0.001	0.298
C18:3 n-3	1.158	0.816	0.059	<0.05
C18:3 n-6	0.198	0.219	0.007	0.168
C20:0	0.146	0.125	0.005	<0.05
cis-11 C20:1	0.501	0.347	0.019	<0.001
C20:2 n-6	0.553	0.523	0.025	0.682
C20:3 n-6	0.774	0.984	0.044	0.074
C20:3 n-3	0.122	0.114	0.006	0.599
C20:4 n-6 (ARA)	2.520	4.375	0.195	<0.001
C20:5 n-3 (EPA)	0.303	0.358	0.009	<0.05
C21:0	0.222	0.242	0.012	0.506
C22:0	0.037	0.061	0.004	<0.01
cis-13 C22:1	0.136	0.161	0.007	0.072
C22:2 n-6	0.071	0.054	0.006	0.209
C22:5 n-3 (DPA)	0.641	1.028	0.063	<0.05
C22:6 n-3 (DHA)	0.413	0.796	0.046	<0.001
C23:0	0.080	0.101	0.006	0.120
C24:0	0.070	0.089	0.005	<0.05
cis-15 C24:1	0.072	0.073	0.006	0.933
Total SFAs	36.139	36.459	0.273	0.604
Total MUFAs	40.317	34.721	0.609	<0.001
Total PUFAs	23.549	28.821	0.597	<0.001
Total UFAs	63.861	63.541	0.273	0.604
Total n-3	2.636	3.111	0.106	0.100
Total n-6	20.885	25.686	0.534	<0.001
C18:0 Δ-9 desaturation index ^2^	0.778	0.739	0.006	<0.05
ARA/(LA + ARA)	0.133	0.180	0.007	<0.001
DHA/(ALA + DHA)	0.271	0.499	0.026	<0.001

^1^ Coeluted with trans-10 C18:1. ^2^ Calculated as cis-9 C18:1/(C18:0 + cis-9 C18:1). ARA: arachidonic acid; EPA: eicosapentaenoic acid; DPA: docosapentaenoic acid; DHA: docosahexaenoic acid; SFAs = saturated fatty acids; MUFAs = monounsaturated fatty acids; PUFAs = polyunsaturated fatty acids; UFAs = unsaturated fatty acids; n-3: omega-3 fatty acids; n-6: omega-6 fatty acids; SEM: standard error of the mean.

**Table 4:** Nutritional fatty acid indexes ^1^ of retail chicken breast meat by label-defined group (NC: non-certified; C: certified).

Parameters	NC	C	SEM	*p*
PUFAs/SFAs	0.66	0.79	0.018	<0.05
n-6/n-3	8.10	8.60	0.240	0.375
LA/ALA	15.35	27.74	1.579	<0.001
EPA + DHA	0.72	1.15	0.051	<0.001
Unsaturation index	99.18	110.58	2.147	<0.001
Nutrition value index	1.86	1.71	0.023	<0.05
Index of atherogenicity	0.43	0.42	0.006	0.288
Index of thrombogenicity	0.91	0.89	0.013	0.584
Hypo- to hypercholesterolemic FAs	2.34	2.40	0.033	0.438
Health-promoting index	2.35	2.42	0.035	0.391
Meat lipid quality	1.99	3.17	0.141	<0.001
Healthy fatty index 1	1.76	1.99	0.026	<0.001
Healthy fatty index 2	2.91	3.14	0.037	<0.05

^1^ Calculated according to Dal Bosco et al. [39]. PUFAs = polyunsaturated fatty acids; SFAs = saturated fatty acids; n-3: omega-3 fatty acids; n-6: omega-6 fatty acids; LA: linoleic acid; ALA: α-linolenic acid; EPA: eicosapentaenoic acid; DHA: docosahexaenoic acid; FAs: fatty acids; SEM: standard error of the mean.

**Table 5:** Average relative abundance (area units × 10^9^) of selected volatile organic compounds (VOCs) of retail chicken breast meat by label-defined group (NC: non-certified; C: certified).

VOCs	RT	LRI	NC	C	SEM	*p*
Butanone	4.34	902	6.25	2.61	0.61	<0.01
2-Ethylfuran	5.39	960	4.13	1.95	0.50	<0.05
Toluene	7.49	1048	6.85	4.64	0.52	<0.05
Heptanal	11.36	1191	20.07	8.83	2.85	<0.05
Dimethyl trisulfide	16.95	1395	2.09	0.65	0.32	<0.05
2,4-Heptadienal (E, E)	19.88	1506	1.34	0.30	0.21	<0.05
Heptylbenzene	23.16	1640	1.37	2.38	0.19	<0.01
4-Ethylbenzoic acid, 2-butylester	26.06	1762	1.69	0.93	0.18	<0.05
2-Pentyl methyl thiazolidine	27.95	1852	1.25	0.64	0.11	<0.01
Pulegol	28.41	1876	1.00	0.41	0.09	<0.001
Trimenal	31.21	2007	0.98	3.86	0.55	<0.01

RT: Retention time. LRI: Linear retention index. SEM: Standard error of the mean.

## Data Availability

The original contributions presented in the study are included in the article. Further inquiries can be directed to the corresponding author.
